# Acetal-Linked Paclitaxel Polymeric Prodrug Based on Functionalized mPEG-PCL Diblock Polymer for pH-Triggered Drug Delivery

**DOI:** 10.3390/polym9120698

**Published:** 2017-12-11

**Authors:** Yinglei Zhai, Xing Zhou, Lina Jia, Chao Ma, Ronghua Song, Yanhao Deng, Xueyao Hu, Wei Sun

**Affiliations:** 1 Department of Biomedical Engineering, School of Medical Devices, Shenyang Pharmaceutical University, Shenyang 110016, China; zyllll1001@126.com (Y.Z.); srh.123@163.com (R.S.); dengyanhao88@163.com (Y.D.); huxueyaosyphu@163.com (X.H.); 2 State Key Laboratory for Marine Corrosion and Protection, Luoyang Ship Material Research Institute (LSMRI), Qingdao 266101, China; 3 Hainan Institute of Materia Medica, Haikou 570311, China; beyondyme@163.com; 4 Department of Pharmacology, School of Life Science and Biopharmaceutics, Shenyang Pharmaceutical University, Shenyang 110016, China; frankjln@126.com; 5 College of Food & Pharmaceutical Engineering, Guizhou Institute of Technology, Guizhou 550003, China; 13765046535@163.com

**Keywords:** prodrug, polymer micelles, pH-sensitive, acetal, paclitaxel

## Abstract

The differences in micro-environment between cancer cells and the normal ones offer the possibility to develop stimuli-responsive drug-delivery systems for overcoming the drawbacks in the clinical use of anticancer drugs, such as paclitaxel, doxorubicin, and etc. Hence, we developed a novel endosomal pH-sensitive paclitaxel (PTX) prodrug micelles based on functionalized poly(ethylene glycol)-poly(ε-caprolactone) (mPEG-PCL) diblock polymer with an acid-cleavable acetal (Ace) linkage (mPEG-PCL-Ace-PTX). The mPEG-PCL-Ace-PTX_5_ with a high drug content of 23.5 wt % was self-assembled in phosphate buffer (pH 7.4, 10 mM) into nanosized micelles with an average diameter of 68.5 nm. The in vitro release studies demonstrated that mPEG-PCL-Ace-PTX_5_ micelles was highly pH-sensitive, in which 16.8%, 32.8%, and 48.2% of parent free PTX was released from mPEG-PCL-Ace-PTX_5_ micelles in 48 h at pH 7.4, 6.0, and 5.0, respectively. Thiazolyl Blue Tetrazolium Bromide (MTT) assays suggested that the pH-sensitive PTX prodrug micelles displayed higher therapeutic efficacy against MCF-7 cells compared with free PTX. Therefore, the PTX prodrug micelles with acetal bond may offer a promising strategy for cancer therapy.

## 1. Introduction

Cytotoxic chemotherapeutics, which act by killing rapidly proliferating cancer cells, remains to be one of the most preferred approaches for treatment of various cancers. However, its application suffers some major limitations such as organ damage, infertility, immunosuppression, and nausea/vomiting which severely reduce patient quality of life [1,2]. Furthermore, many anticancer drugs have low aqueous solubility, which requires the use of organic solvents or surfactants. These drawbacks therefore urge scientists to develop improved chemotherapy formulations by which the use of solubilizer would no more be needed and the selectivity and efficacies toward cancer cells can be enhanced [3].

In the past decades, nanoparticles based on enhanced permeability and retention (EPR) effect such as micelles [4,5], liposomes [6,7], and dendrimers [8,9] have been intensively reported as drug delivery systems. Compared with free drugs, chemotherapeutics conjugated with nanoparticles have the advantages of passive selective accumulation at the cancer tissues and increase the solubility of drug molecules [10,11,12]. Among them, responsive polymeric nanoparticles have emerged as one of the most promising delivery systems for anticancer drugs [13,14,15]. Internal and external stimuli, such as temperature [16,17,18,19], pH [20,21,22,23,24], or enzyme [25,26,27,28] were applied to maximal drug release in target tissues. As reported, the extracellular of solid tumors (pH 6.0–7.0) is slightly acidic compared with normal tissues, and pH inside cancer cells is even lower, especially inside endosome (pH 5.0–6.0) and lysosome (pH 4.0–5.0) [29,30]. Based on this unusual tumor tissue environment, a great variety of pH responsive polymer prodrugs were designed with acid-labile chemical bonds such as acetal [31], imine [32], hydrozone [33], and so forth.

Acetals are promising candidates for the development of acid-sensitive connections with hydroxyl groups because the rate of hydrolysis is highly relative to the concentration of hydronium ion [34]. To the best of my knowledge, several groups developed pH sensitive polymeric prodrugs via acetal bond. However, most of them show the disadvantages such as non-biodegradable or low drug content [31,35,36].

Poly(caprolactone) (PCL) is one of the extensively employed aliphatic polyestesr due to its excellent biocompatibility and biodegradability. Owing to the appropriate hydrophilic and hydrophobic balance, amphiphilic di-block copolymers based on poly(ethylene glycol)-block-poly(caprolactone) (PEG-b-PCL) show great potential to delivering water-insoluble hydrophobic drugs due to their large solubilizing power and loading capacity [37,38,39]. However, these copolymers are not suitable for the preparation of pH sensitive anti-cancer prodrug because of the absence of functional groups. Surnar and Jayakannan reported a newly developed pH-responsive PEG-b-PCL with carboxylic groups, which makes the synthesis of an acid-labile anti-cancer prodrug possible [40].

In this work, we report acetal-linked PTX prodrugs with different PTX content. The prodrug micelles offer two advantages: (i) the pH sensitive acetal bond offers prodrug micelles possessing specific tumor targetability. Moreover, the firmer conjugation between PEG-b-PCL and PTX may reduce the undesired drug release during circulation in the bloodstream; and (ii) the superior drug content can be easily achieved by varying PTX-to-polymer molar ratio. The structure and physiochemical characteristics of PTX prodrug and its micelles were determined using ^1^H NMR, gel permeation chromatography (GPC), dynamic light scattering (DLS) and transmission electron microscopy (TEM). We also investigated pH-dependent drug release from PTX prodrug micelles and in vitro cytotoxicity of PTX-conjugated micelles to MCF-7 cell.

## 2. Experimental Section

### 2.1. Materials

1,4-Cyclohexanediol, potassium *t*-butoxide, *t*-butyl acrylate, pyridinium chlorochromate (PCC), metachloroperbenzoic acid (MCPBA), tin (II) 2-ethylhexanoate (Sn(Oct)_2_), trifluoroacetic acid (TFA), Dicyclohexylcarbodiimide (DCC), and 4-dimethylaminopyridine (DMAP) were purchased from Aladdin Industrial Corporation (Shanghai, China). Paclitaxel (PTX) and Molecular sieves (4 Å) were obtained from Meilun Biotechnology Co., Ltd. (Dalian, China). Polyethylene glycol monomethyl ether (MW = 5000, here after referred as mPEG, Sigma-Aldrich, Shanghai, China), 2-(ethenyloxy) ethanol (Nanjing Chemlin, Nanjing, China) and *p*-toluenesulfonic acid monohydrate (*p*-TSA, Acros, Beijing, China) were used directly without any purification. *N*,*N*-dimethylformamide (DMF) was firstly dried by MgSO_4_ and consequently distilled under reduced pressure, and 1,4-dioxane underwent the process of refluxing with sodium wire and distilling under nitrogen atmosphere prior to use. All other reagents of analytical grade were used without further purification. Human breast adenocarcinoma cell line (MCF-7 cell) was from American Type Culture Collection (ATCC) (Beijing, China). RPMI Medium 1640, Dulbecco’s Modified Eagle Medium (DMEM) and 0.25% Trypsin-EDTA were purchased from Thermo Fisher Scientific (Shanghai, China). Fetal Bovine Serum was obtained from Biological Industries (Shanghai, China).

### 2.2. Synthesis of Carboxylic Functionalized mPEG-PCL (mPEG-CPCL)

All steps for the synthesis of the carboxylic functionalized mPEG-PCL (mPEG-CPCL) were carried out as reported previously [40] but modifying some certain reactions’ conditions, for example, the addition amount of some reagents, the lasting time of some reactions, et al.

#### 2.2.1. Synthesis of 3-(4-Hydroxy-cyclohexyloxy)-propionic Acid *t*-Butyl Ester (1)

To the stirred solution of 1,4-cyclohexanediol (10.0 g, 86.0 mmol) in THF (150 mL), potassium *t*-butoxide (10 g, 89.1 mmol) was added portion wise, and the mixture was stirred for 30 min under nitrogen protection. Subsequently, *t*-butyl acrylate (5.5 g, 43.0 mmol) in THF (25 mL) was added slowly by using a dropping funnel, and then the reaction mixture was refluxed under nitrogen protection for another 48 h. The solvent was evaporated under reduced pressure, and the content was neutralized with 1 N HCl (10 mL). It was extracted with ethyl acetate, and the organic layer was dried over anhydrous MgSO_4_. The solvent was concentrated and the residue was further purified by silica gel column chromatography using ethyl acetate and hexane (1:5 *v*/*v*) as eluent. Yield: 5.4 g (51.4%). ^1^H NMR (600MHz, CDCl_3_) δ 3.64 (m, 3H, O–CH_2_– and CHO), 3.27 (m, 1H, CH–OH), 2.44 (t, 2H, –CH_2_CO–), 1.96 (m, 2H, –CH(CH_2_)_2_), 1.79 (m, 2H, –CH(CH_2_)_2_), 1.64–1.32 (m, 4H, –CH(CH_2_)_2_), 1.45 (s, 9H, –C(CH_3_)_3_) (Supplementary Materials, Figure S1).

#### 2.2.2. Synthesis of 3-(4-Oxo-cyclohexyloxy)-propionic Acid *t*-Butyl Ester (2)

PCC (18.8 g, 87.4 mmol) was added to the solution of compound **1** (10.7 g, 43.7 mmol) in DCM (200 mL) under nitrogen protection and then the reaction mixture was stirred at room temperature overnight. The reaction mixture was filtered through a Buchner funnel with the celite to remove PCC salts. The filtrate was concentrated, and the residue was purified by silica gel column chromatography using ethyl acetate and hexane (1:4 *v*/*v*) as eluent. Yield: 10.2 g (96%). ^1^H NMR (600 MHz, CDCl_3_) δ 3.70 (m, 3H, O–CH_2_ and O–CH), 2.55 (m, 2H, CO–CH_2_), 2.49 (t, 2H, CO–CH_2_), 2.22 (m, 2H, CO–CH_2_), 2.08 (m, 2H, CH(CH_2_)), 1.87 (m, 2H, CH(CH_2_)), 1.45 (s, 9H, C(CH_3_)_3_) (Supplementary Materials, Figure S2).

#### 2.2.3. Synthesis of 3-(7-Oxo-oxepan-4-yloxy)-propionic Acid *t*-Butyl Ester (3)

To a stirred solution of compound **2** (10.5 g, 43.3 mmol) in DCM (100 mL), 75% metachloroperbenzoic acid (12.0 g, 52.2 mmol) was added slowly at 0 °C. Subsequently, anhydrous NaHCO_3_ (10.9 g, 130.0 mmol) was added to the above reaction mixture, and the reaction mixture was allowed to warm to room temperature and stirred for another 12 h. The reaction was diluted with DCM (50 mL) and quenched by the addition of solid sodium thiosulphate (9.3 g, 58.8 mmol). The mixture was stirred for 1 h, filtered and the solid residues were washed with DCM (50 mL). The filtrate was evaporated under reduced pressure and the crude product was purified by silica gel column chromatography using ethyl acetate and hexane (1:5 *v*/*v*) as eluent. Yield: 10.1 g (90%). ^1^H NMR (600 MHz, CDCl_3_) δ 4.45 (m, 1H, COOCH_2_), 4.02 (m, 1H, COOCH_2_), 3.63 (m, 3H, OCH_2_ and OCH), 2.94 (t, 1H, CH_2_CO), 2.46 (t, 2H, COCH_2_), 2.38 (m, 1H, CH_2_CO), 1.99 (m, 2H, OCH–(CH_2_)_2_), 1.90 (t, 1H, OCH–(CH_2_)_2_), 1.78 (t, 1H, OCH–(CH_2_)_2_),1.45 (s, 9H, C(CH_3_)_3_) (Supplementary Materials, Figure S3).

#### 2.2.4. Synthesis of mPEG-Bupcl Diblock Polymer

mPEG-BuPCL was synthesized by ring-opening polymerization (ROP). Initiator mPEG5000 (400 mg, 0.08 mmol), substituted caprolactone monomer compound **3** (1032 mg, 4 mmol) and Sn(Oct)_2_ (16.2 mg, 0.04 mmol) were taken in a dry Schlenk flask. After vacuumed and charged in nitrogen, the reaction mixture was immersed in preheated oil bath at 130 °C, and the polymerization was continued for 48 h with constant stirring. After cooling to room temperature, DCM (20 mL) was added to the sticky polymer solution. The polymer was purified by pouring into icediethyl ether and then filtered to gain the off-white viscous solid of mPEG-BuPCL 930 mg (conversion rate: 80%). ^1^H NMR (600 MHz, CDCl_3_) δ 4.11 (m, OCH_2_), 3.66–3.63 (m, PEG and OCH_2_), 3.44 (m, OCH), 2.43 (t, COCH_2_), 2.36 (m, COCH_2_), 1.90–1.73 (m, CH(CH_2_)_2_), 1.45 (s, C(CH_3_)_3_) (Supplementary Materials, Figure S4) .

#### 2.2.5. Synthesis of mPEG-CPCL

Trifluoroacetic acid (5.2 mL) was added dropwise into the solution of mPEG-BuPCL (2.7 g) in DCM at 0 °C. After finishing the addition, the reaction mixture was allowed to warm to room temperature and stirred for 8 h. The solvents were evaporated, and the polymer was redissolved in DCM (5 mL) and precipitated in freezing-cold ether. The purification was repeated at least twice to get pure mPEG-CPCL 2.2 g (yield: 96%). ^1^H NMR (600 MHz, CDCl_3_) δ 4.15 (s, OCH_2_), 3.71–3.65 (d, PEG and OCH_2_), 3.50 (s, OCH), 2.58 (s, COCH_2_), 2.39 (s, COCH_2_), 1.88–1.79 (d, CH(CH_2_)_2_).

### 2.3. Synthesis of PTX Prodrug (mPEG-PCL-Ace-PTX)

#### 2.3.1. Synthesis of Vinyl Ether-Functionalized mPEG-PCL (mPEG-VPCL)

To the solution of mPEG-CPCL (242 mg, equiv. 0.74 mmol COOH) in anhydrous 1,4-dioxane (10 mL), DMAP (90 mg, 0.74 mmol) and DCC (458 mg, 2.22 mmol) were added at 0 °C, respectively. Subsequently, the flask was vacuumed and charged in nitrogen gas. The reaction mixture was allowed to warm to room temperature and proceeded under stirring for 24 h at 25 °C. Thereafter, 2-(ethenyloxy) ethanol (664 μL, 7.4 mmol) was added and the mixture continued stirring for another 72 h at 25 °C in the darkness. The cloudy solution was filtered, and the filtrate was purified by extensive dialysis against 1000 mL DI water with 10 changes of water for 48 h and collected by freeze-drying. Yield: (148 mg, 58%). ^1^H NMR (600 MHz, CDCl_3_) δ 6.45 (q, CH=CH_2_), 4.32 (t, OCH_2_), 4.20–4.18 (dd, CH=CH_2_), 4.12 (m, OCH_2_), 4.03 (dd, CH=CH_2_), 3.88 (t, OCH_2_), 3.71–3.63 (m, PEG and OCH_2_), 3.45 (s, OCH), 2.56 (t, COCH_2_), 2.34 (t, COCH_2_), 1.86–1.76 (m, CH(CH_2_)_2_).

#### 2.3.2. Synthesis of mPEG-PCL-Ace-PTX

mPEG-PCL-Ace-PTX was synthesized by using the procedure described below. To a 25 mL Schlenk flask, mPEG-VPCL (150 mg, equiv. 119.0 μmol –CH=CH_2_), PTX (51 mg, 59.5 μmol), *p*-TSA (1.5 mg, catalytic amount) and 4 Ǻ molecular sieve (1 g) were added into anhydrous DMF (5 mL) under a nitrogen flow. The flask was vacuumed and sealed with parafilm. Then the reaction was carried out under stirring at 25 °C. After 5 days, the mixture passed through a Buchner funnel and the filtrate was collected and dialyzed against 500 mL DMF (MWCO = 3500 Da) for 24 h with 4 times change of DMF to remove the unconjugated PTX, and then the resulting mixture was further dialyzed against 1000 mL DI water with 10 times change of water for another 48 h, and then the desired product was collected by freeze-drying. Yield: (147 mg, 75%). ^1^H NMR (600 MHz, CDCl_3_) PTX (δ 8.12, 7.74, 7.60, 7.52–7.47, 7.42–7.38, 7.33, 7.07, 6.26, 6.21, 5.77, 5.66, 4.93, 4.79, 3.79, 2.58–2.53, 2.38–2.23, 1.90–1.76, 1.68, 1.24, 1.14), acetal bond (–OCH(CH_3_)O–: δ 4.38; –OCH(CH_3_)O–: δ 1.24), 2-(ethenyloxy) ethyl (CH=CH_2_: δ 6.45, 4.18, 4.03; –OCH_2_CH_2_O–: δ 4.32, 3.88) and PEG-CPCL (δ 4.12, 3.45, 3.70–3.64, 2.56, 2.35 and 1.90–1.76).

### 2.4. Structural Characterization

All compounds and polymers obtained at each step were characterized by ^1^H NMR. A Bruker AVANCE III HD instrument equipped with a 600 MHz magnetic field was employed to record ^1^H NMR spectra. The proton signals of all the products were measured in CDCl_3_ solution. The ${\bar{M}}_{n}$ of conjugates and PTX loading contents in the prodrug were determined by the characteristic peaks of PEG, PCL and PTX.

The number-averaged molecular weight (${\bar{M}}_{n}$) and molecular weight distribution (PDI) was measured by a gel permeation chromatography (Waters GPC system, Shanghai, China) equipped with 1515 isocratic HPLC pump (Waters Corp, Shanghai, China), 2414 Refractometer Detector and a GPC column (Waters Styragel HT3 THF, Shanghai, China).The polymers were dissolved in THF at a concentration of 5 mg/mL. All tests were carried out at ambient temperature using THF as eluent at a flow rate of 1 mL/min and the polystyrene as standards for calibration.

### 2.5. Preparation of mPEG-PCL-Ace-PTX Micelles

The polymer prodrug was dissolved in THF, and then the resultant solution was dialyzed against phosphate buffer (pH 7.4, 10 mM) (MWCO = 3500 Da). After 24 h, the water phase in dialysis bag was centrifuged and further purified by passing through 0.22 μm-microfiltration membrane. Subsequently, it was lyophilized and stored in a dry environment.

### 2.6. Particle Size and Surface Morphology Assessment

Dynamic light scattering was employed to determine the hydrodynamic diameters of mPEG-PCL-Ace-PTX micelles. In brief, 10 mg lyophilized mPEG-PCL-Ace-PTX micellar sample were dissolved in 10 mL Millipore water followed by ultrasonic treatment to obtain the uniform micelles dispersion. Both the hydrodynamic size and zeta potential of micelles were evaluated by ZetasizerNano ZS (MalvernInstruments) at 25 °C (*n* = 3).

Likewise, aqueous micellar solution (1 mg/mL) was put dropwise onto the clean copper grids and dried in the air; the size and morphology of micelles was determined by transmission electron microscopy (TEM) (JEOL JEM-100CX II, Tokyo, Japan). The image was calculated with reference to the ruler for getting micelle core size and size distribution.

### 2.7. In Vitro Drug Release

The micelle solution (1 mL) in a dialysis bag (MWCO = 3500 Da) was suspended in three different media, i.e., pH 5.0 (acetate buffer), pH 6.0 (acetate buffer) and pH 7.4 (phosphate buffer). The in vitro release was carried out under the sink condition. Temperature was held constant at 37 °C throughout the experiments. 3 mL of the release medium was withdrawn and replenished with 3 mL of fresh media at designated intervals. The amount of PTX released was analyzed by Shimadzu UV-2100 spectrophotometer (λ_max_ = 228 nm).

### 2.8. In Vitro Cellular Uptake

The polymer prodrug and coumarin-6 were co-dissolved in THF, and then the formed solution was transferred into a dialysis bag (MWCO = 3500 Da) against phosphate buffer (pH 7.4, 10 mM). After 24 h, the water solution in dialysis bag was centrifuged to obtain a supernatant which was further purified by passing through 0.22 μm filter. Then it was lyophilized and stored in a dry environment.

MCF-7 cells were seeded in 24-well plates at a density of 5 × 10^4^ cells per well in DMEM medium and incubated at 37 °C under a 5% CO_2_ atmosphere for 24 h. Then the cells were incubated with micelles encapsulating coumarin-6 for 1, 2 and 4 h. The MCF-7 cells were washed three times with PBS and fixed with 100% ethanol for 10 min at room temperature. The cell nuclei were stained with Hoechst 33342. Finally, the cells were monitored by Nikon C3 confocal laser scanning microscope.

### 2.9. In Vitro Cellular Viability

The cytotoxicity of PTX prodrug nanoparticles against human breast cancer cells (MCF-7) was studied using the MTT assay. MCF-7 cells were seeded in a 96-well plate (5 × 10^3^) and incubated for 24 h. The media was aspirated and charged with fresh media containing various concentrations of PTX prodrug micelles. The MCF-7 cells were cultured in an atmosphere containing 5% CO_2_ at 37 °C for 48 h. Then, 10 μL of MTT solution (5 mg/mL) was added. The MCF-7 cells were incubated for another 4 h. After discarding the culture medium, the MTT-formazan generated by live cells was dissolved in 100 μL of DMSO, and the absorbance at a wavelength of 492 nm of each well was recorded using a microplate reader.

## 3. Results and Discussion

### 3.1. Synthesis and Characterization of mPEG-PCL-Ace-PTX

**Scheme 1:**
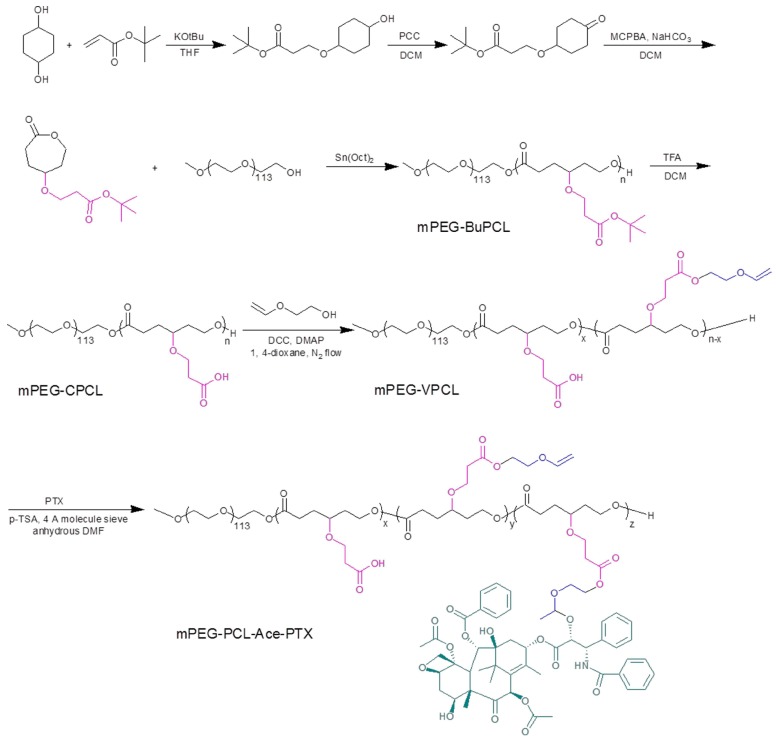
Synthesis of mPEG-CPCL and mPEG-PCL-Ace-PTX.

In order to acquire a functionalized mPEG-PCL diblock polymer with pH-sensitive acetal groups, we employed a new functional caprolactone monomer with a protected carboxyl group as reported [40]. According to the synthetic route shown in Scheme 1, substituted caprolactone monomer compound **3** was synthesized in modest yield by using commercially available 1,4-cyclohexanediol as the starting material via Michael addition, PCC oxidation, and Baeyer-Villiger oxidation, respectively. All of the above intermediates were characterized by ^1^H NMR. Subsequently, compound **3** was subjected to the ROP employing mPEG 5000 as macroinitiator. From ^1^H NMR spectra of mPEG-BuPCL (Supplementary Materials, Figure S4), it showed clearly characteristic peaks of PEG (δ 3.63) and BuPCL (δ 1.45, 1.7–1.9, 2.3–2.5, 3.44, 3.6–3.7, and 4.11).

**Figure 1:**
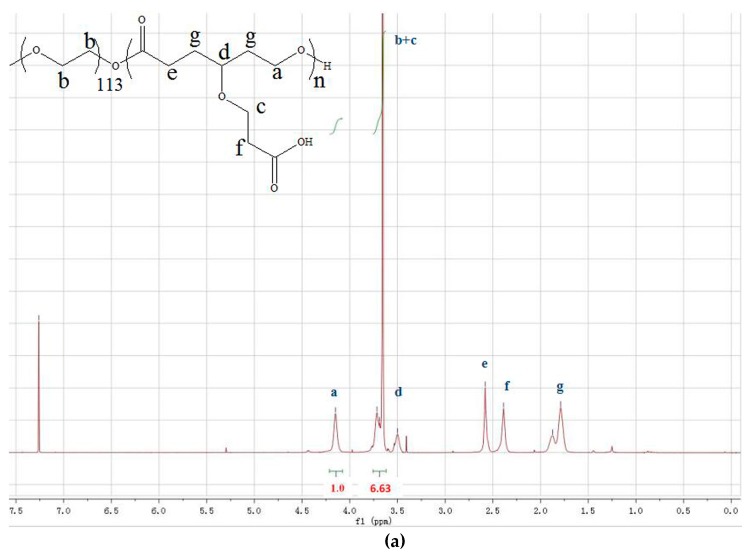

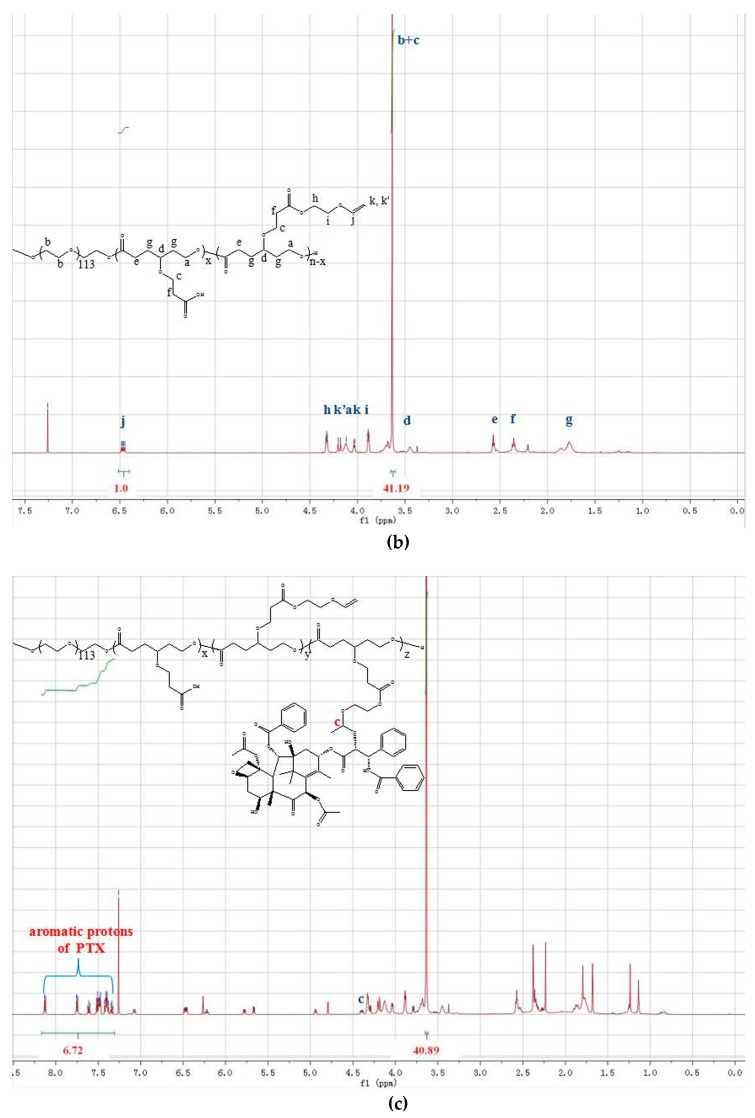
^1^H NMR spectra of mPEG-CPCL (**a**), mPEG-VPCL (**b**) and mPEG-PCL-Ace-PTX (**c**) in CDCl_3_.

The degree of polymerization of BuPCL block was estimated by comparing the intensities of signals at δ 4.11 and 3.63 to be 40. The *t*-butyl ester groups in the copolymer of mPEG-BuPCL were hydrolyzed by excessive trifluoroacetic acid to produce the corresponding carboxylic acid derivatives mPEG-CPCL. The peak assigned to be the methyl protons contributing to the *tert*-butyl groups at δ 1.45 ppm disappeared, which demonstrates the full hydrolysis of all the *tert*-butyl groups (Figure 1a). 2-(Ethenyloxy) ethanol was coupled with mPEG-CPCL employing DCC and DMAP in 1,4-dioxane for 72 h to acquire mPEG-VPCL. ^1^H NMR spectra showed new peaks at δ 6.45/4.18/4.03 and δ 4.32/3.88 rather than peaks of mPEG-CPCL (Figure 1b). The number of vinyl ether coupled with per polymer chain was calculated to be 11 by comparing integrals of peaks at δ 6.45 with δ 3.63.

Finally, the acid-labile polymeric prodrug was obtained through a “click”-type conjugate reaction between 2′-hydroxyl group of PTX and the pendent vinyl ethers of mPEG-VPCL as reported before [31]. According to ^1^H NMR (Figure 1c), it clearly showed the peaks attributed to PTX at δ 1.1–1.3, 1.6–1.9, 2.2–2.6, 4.7–5.0, 5.6–5.8, 6.2–6.3, and 7.0–8.2 besides the signals of mPEG-VPCL. Meanwhile, a peak assigned to acetal methine proton appeared at δ 4.38, which strongly supported the conjugation between PTX and mPEG-PCL via an acetal bond. The PTX content of prodrugs was determined according to the ^1^H NMR spectra by comparing integrals of peaks at δ 7.0–8.2 with δ 3.64, which indicated that PTX prodrugs with 15.6 and 23.5 wt % PTX obtained by varying PTX-to-polymer molar ratios of 2/1 and 3/1. Notably, these PTX prodrugs have significantly higher drug contents compared with PTX-acetal-pDMA [35] and PTX-acetal-PEG without free PTX encapsulated [36] (only one molecular of PTX was chemically conjugated to the end of the polymers). GPC measurement showed a monomodal distribution of PTX prodrugs with a low polydispersity index (PDI) of 1.16 and 1.10 (Table 1, Supplementary Materials, Figure S5).

### 3.2. Pharmaceutical Evaluation of mPEG-PCL-Ace-PTX Micelles

**Figure 2:**
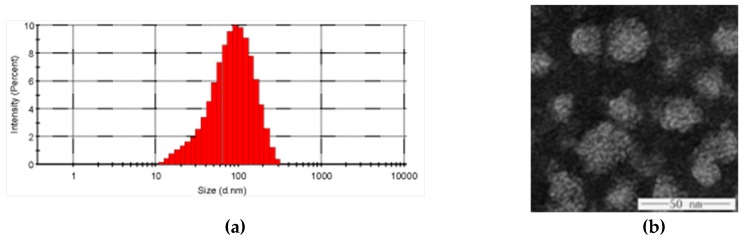
Size distribution profiles for mPEG-PCL-Ace-PTX_5_ micelles determined by dynamic light scattering (DLS) (**a**) and transmission electron microscopy (TEM) (**b**).

Due to the amphiphilic property, mPEG-PCL-Ace-PTX could self-assemble into nanosized micelles by dialyzing against phosphate buffer (pH 7.4, 10 mM), in which hydrophobic polyester and hydrophilic PEG form the core and the shell, respectively. Based on the higher PTX content, the size distribution and morphology of the mPEG-PCL-Ace-PTX_5_ micelles was determined by DLS and TEM. The DLS measurement result showed the average particle size of mPEG-PCL-Ace-PTX_5_ to be 68.5 nm (Figure 2a). TEM image indicated that the mPEG-PCL-Ace-PTX_5_ micelles were well-dispersed in aqueous solution in the form of spherical morphology with an average diameter of about 50 nm (Figure 2b), which is somewhat smaller than that determined by DLS, likely due to shrinkage of the micelles upon drying.

**Figure 3:**
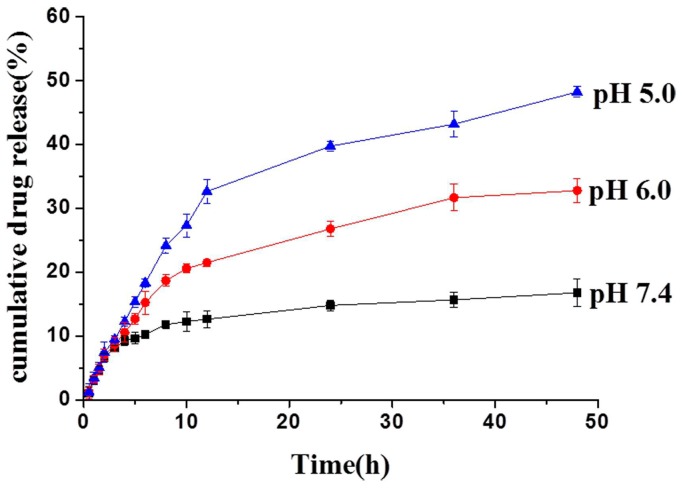
pH-dependent PTX release from mPEG-PCL-Ace-PTX_5_ micelles. The data were presented as mean ± standard deviation (*n* = 3).

Because PTX was covalently conjugated to the mPEG-VPCL di-block copolymers through a pH-sensitive acetal linkage, the mPEG-PCL-Ace-PTX_5_ micelles are expected to be pH-responsive. The in vitro drug release from the mPEG-PCL-Ace-PTX_5_ micelles was investigated at 37 °C under three different pH levels, i.e., pH 5.0, 6.0, and 7.4. As shown in Figure 3, the cumulative release of PTX was 16.8%, 32.8%, and 48.2% in 48 h at pH 7.4, 6.0, and 5.0, respectively, which demonstrated that PTX release was remarkably accelerated under moderate acid environments. It should be noted that the drug release ratio of mPEG-PCL-Ace-PTX5 micelles is relatively lower than that of PEG-PAA-acetal-PTX [31] and PEG-acetal-PTX with free PTX encapsulated in 48 h at pH 7.4 [36]. However, a comparatively higher PTX release was observed for mPEG-PCL-Ace-PTX5 micelles compared with PTX-acetal-pDMA in 48 h under a mildly acidic environment. Moreover, as expected, no burst release was observed under both normal physiological and acidic conditions due to the covalent linkage between PTX and mPEG-VPCL. These results demonstrate that mPEG-PCL-Ace-PTX5 micelles possess the characteristics of better stability and lower undesired drug leakage in process of drug delievery.

**Figure 4:**
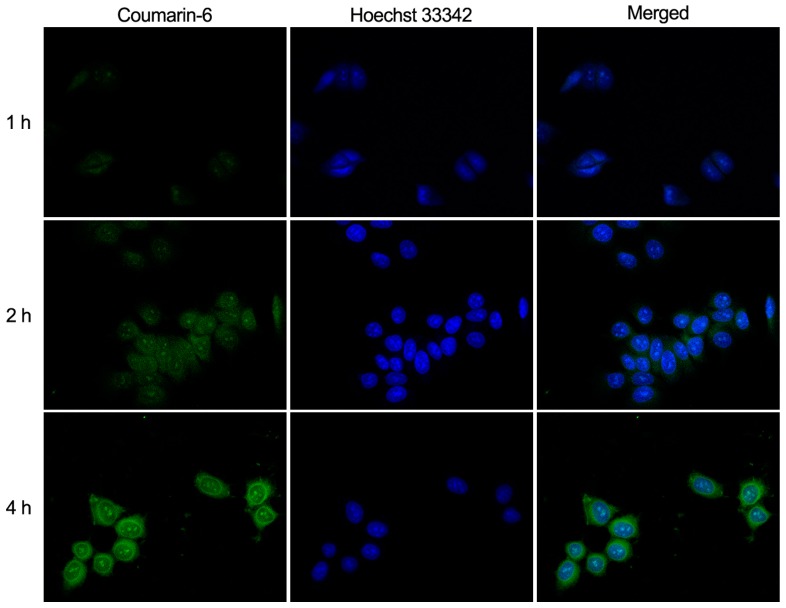
Confocal laser scanning microscopy images of MCF-7 cells incubated with micelles encapsulating coumarin-6 for 1, 2, and 4 h.

In order to investigate the cellular uptake behaviors by MCF-7 cells, confocal laser scanning microscopy was employed. Micelles encapsulating coumarin-6 were incubated for 1, 2, and 4 h, respectively. As shown in Figure 4, after 1 h incubation at 37 °C, the fluorescence intensity in the cytoplasm of MCF-7 cells was relatively very weak. When the incubation period elongated to 2 and 4 h, the quantity of green fluorescence increased dramatically, demonstrating efficient internalization of micelles.

**Figure 5:**
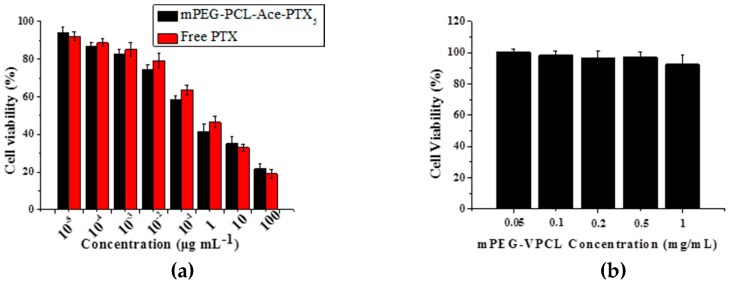
MTT assays of PTX mPEG-PCL-Ace-PTX_5_ micelles (**a**) and Vinyl Ether-Functionalized mPEG-PCL (**b**) against MCF-7 cells. Data are presented as the average ± standard deviation (*n* = 3).

The in vitro anti-tumor activity of PTX prodrug was evaluated employing MTT assay. As shown in Figure 5a, the result indicated that PTX prodrug micelles displayed significant activity of inhibiting proliferation against MCF-7 cells. At drug concentrations lower than 1.0 μg·mL^−1^, drug efficacy of PTX prodrug micelles was similar with free PTX or little higher against MCF-7 cells. In contrast, when drug concentrations was higher than 1.0 μg·mL^−1^, antitumor activity of PTX prodrug micelles became slightly lower compared with free PTX. The half-maximal inhibitory concentrations (IC_50_) were determined to be 0.49 and 0.65 μg·mL^−1^ for PTX prodrug micelles and free PTX, respectively. It is clear that the PTX prodrug micelles shows an improvement of the cytotoxicity compared to the parent free PTX, which contributes to rapid cleavage of acetal linkage in response to the intracellular pH of MCF-7 cells [41,42,43]. 

Biocompatibility is one of the most important factors to be considered in the application of drug delivery system. Hence it is necessary to investigate the safety of the precursor of PTX prodrug. As shown in Figure 5b, vinyl ether-functionalized mPEG-PCL was actually nontoxic to MCF-7 cells, which tested concentrations varied from 0.05 to 1.0 mg/mL. The data of cell viability indicates that vinyl ether-functionalized mPEG-PCL possesses good biocompatibility and is safe as drug nanocarrier.

## 4. Conclusions

In conclusion, we have successfully developed a pH responsive polymer-drug conjugate for PTX delivery based on PEG-PCL, which exhibited excellent in vitro antitumor activity to MCF-7 cells. These smart PTX prodrug nanoparticles possess several unique features: (i) the mPEG-PCL-Ace-PTX micelles have a narrow size distribution and remarkable drug contents (23.5 wt % PTX); (ii) Owing to the acetal linkage between PTX and mPEG-CPCL, the PTX prodrug shows accelerated drug release in tumor-relevant acid micro-environment;(iii) They exhibit improved antitumor activity and good biocompatibility. (iv) They can readily be synthesized with controlled structures and molecular weight from PEG-PCL diblock polymer. All these results demonstrate that these acetal-linked PTX prodrug micelles have appeared as a highly promising alternative for cancer chemotherapy.

## Figures and Tables

**Table 1:** Synthesis of mPEG-PCL-Ace-PTX.

PTX Prodrug	*M*_n_^a^ (kg/mol)	*M*_w_/*M*_n_^b^ (PDI)	PTX ^c^ (wt %)
mPEG-PCL-Ace-PTX_3_	16.4	1.16	15.6
mPEG-PCL-Ace-PTX_5_	18.1	1.10	23.5

^a^ Determined from ^1^H NMR spectrum. ^b^ Determined from GPC measurements. ^c^ Calculated by ^1^H NMR via comparing integrals of signals at δ 7.0–8.2 with δ 3.64.

## Supplementary Materials

The following are available online at www.mdpi.com/2073-4360/9/12/698/s1.
