# Supplementary material for: Acetal-Linked Paclitaxel Polymeric Prodrug Based on Functionalized mPEG-PCL Diblock Polymer for pH-Triggered Drug Delivery
Source: Polymers (Basel). 2017 Dec 11;9(12):698. doi: 10.3390/polym9120698 (PMC6418821; doi:10.3390/polym9120698)
Supplement: Supplementary file 1 [file polymers-09-00698-s001.pdf]

Supplementary Materials

Acetal-linked paclitaxel polymeric prodrug based on functionalized mPEG-PCL diblock polymer for pH-triggered drug delivery

Yinglei Zhai [1, 5,†], Xing Zhou [2,†], Lina Jia [3], Chao Ma [4], Ronghua Song [1], Yanhao Deng [1], Xueyao Hu [1], Wei Sun [1,*]

[1]Department of Biomedical Engineering, School of Medical Devices, Shenyang Pharmaceutical University, Shenyang 110016, China
[2]Hainan Institute of Materia Medica, Haikou 570311, China
[3] Department of Pharmacology, School of Life Science and Biopharmaceutics, Shenyang Pharmaceutical University, Shenyang 110016, China
[4]College of Food & Pharmaceutical Engineering, Guizhou Institute of Technology, Guizhou 550003, China
[5] State Key Laboratory for Marine Corrosion and Protection, Luoyang Ship Material Research Institute (LSMRI), Qingdao, 266101, China
†These two authors contributed equally to this work.

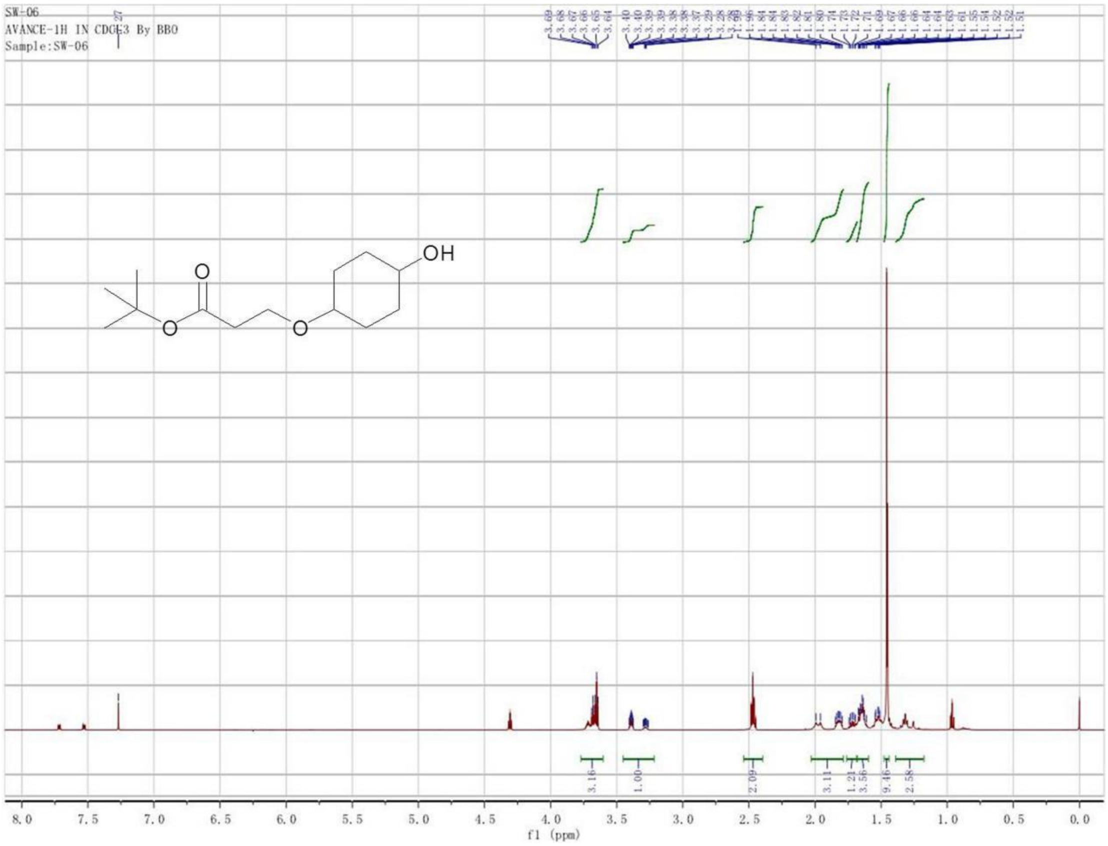

Figure S1. ^{1}H NMR spectra of 3-(4-Hydroxy-cyclohexyloxy)-propionic acid t-butyl ester (1).

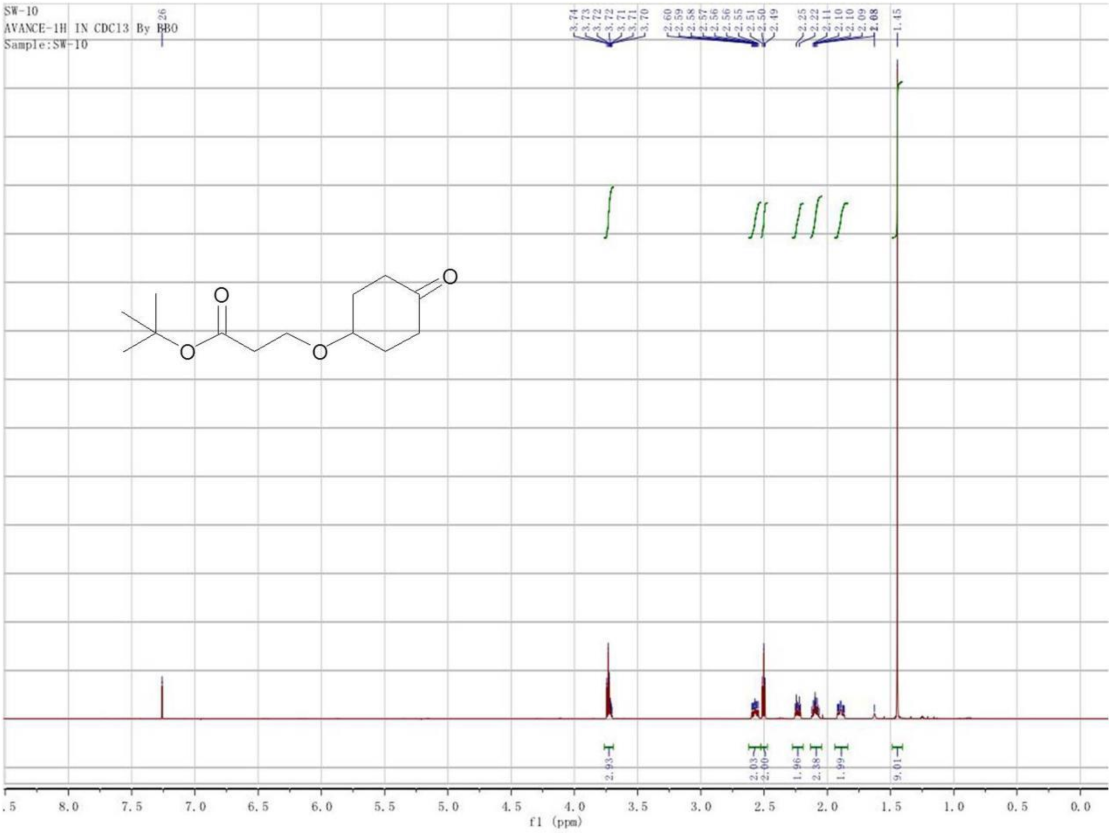

Figure S2. ^{1}H NMR spectra of 3-(4-Oxo-cyclohexyloxy)-propionic acid t-butyl ester (2).

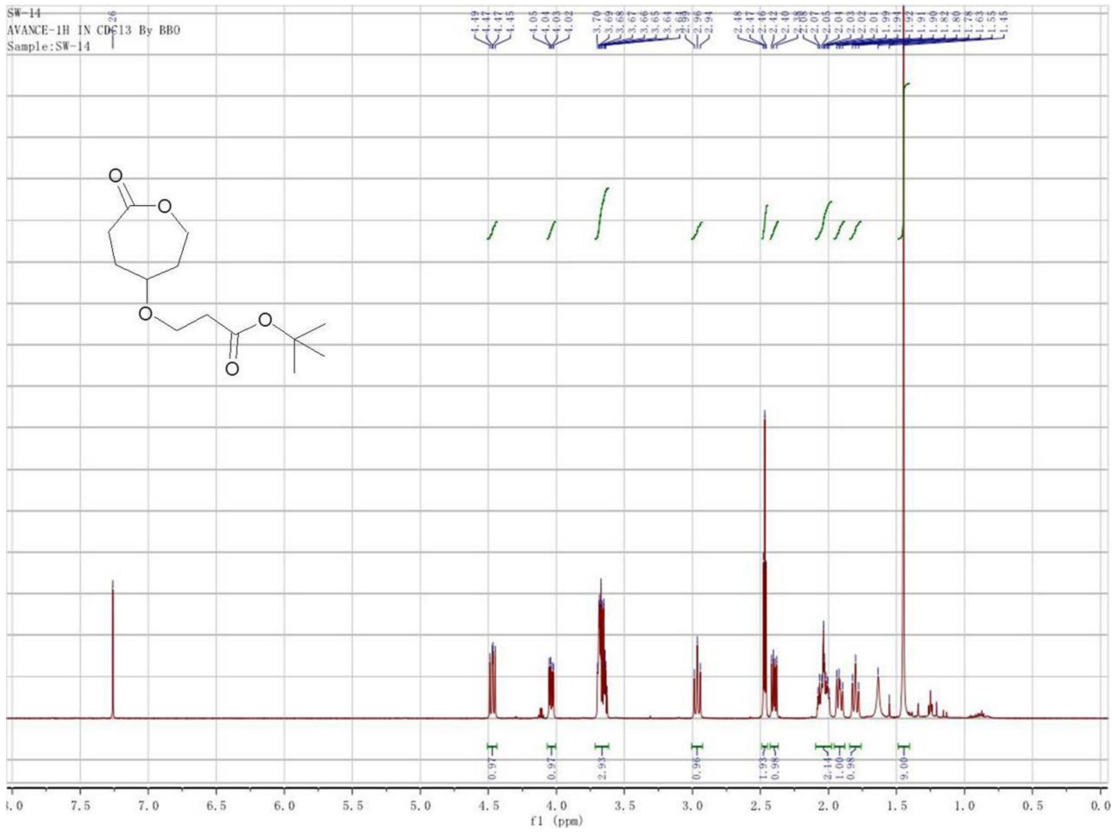

Figure S3. ^{1}H NMR spectra of 3-(7-Oxo-oxepan-4-yloxy)-propionic acid t-butyl ester (3).

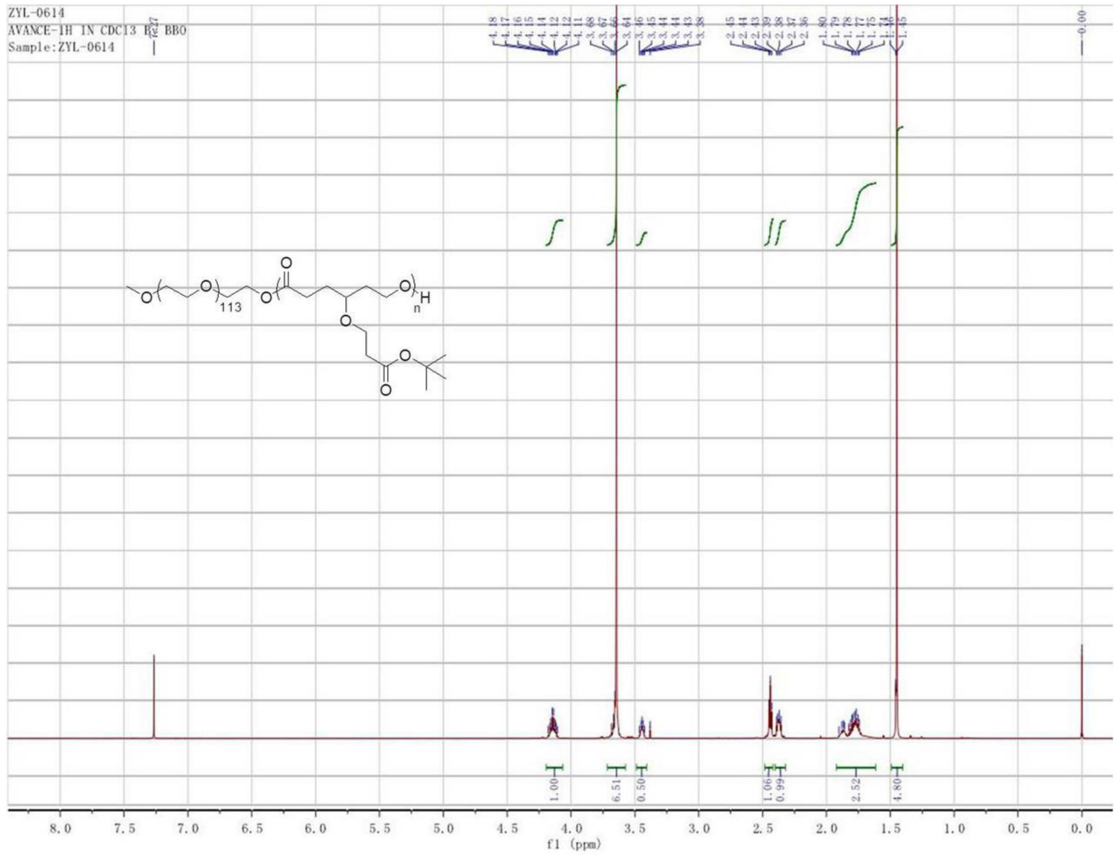

Figure S4. ^{1}H NMR spectra of mPEG-BuPCL Diblock Polymer.

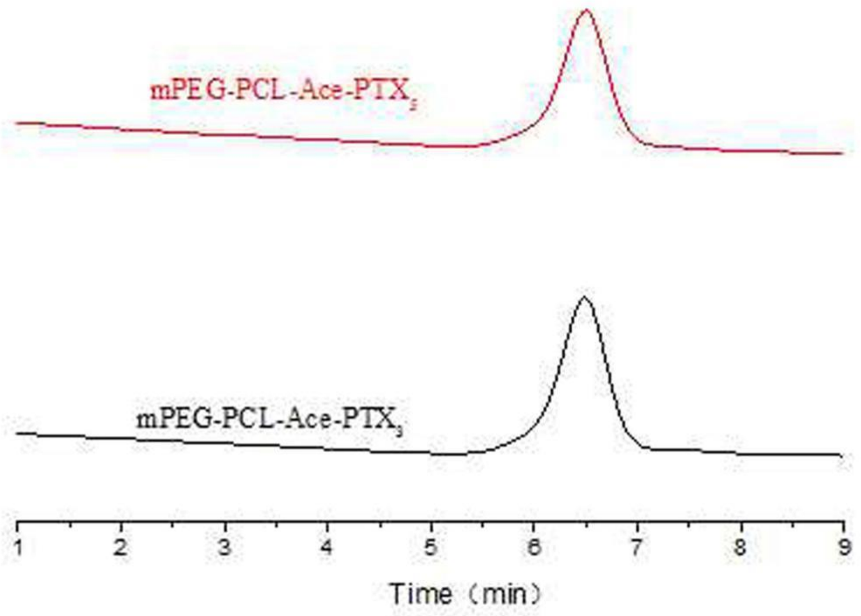

Figure S5. GPC trace of polymers: mPEG-PCL-Ace-PTX_5 and mPEG-PCL-Ace-PTX_3
